# Program for Integration and Rapid Analysis of Mass Isotopomer Distributions (PIRAMID)

**DOI:** 10.1093/bioinformatics/btad661

**Published:** 2023-10-27

**Authors:** Javier D Gomez, Martha L Wall, Mohsin Rahim, Shrikaar Kambhampati, Bradley S Evans, Doug K Allen, Maciek R Antoniewicz, Jamey D Young

**Affiliations:** Department of Chemical and Biomolecular Engineering, Vanderbilt University, Nashville, TN, 37240, United States; Department of Chemical and Biomolecular Engineering, Vanderbilt University, Nashville, TN, 37240, United States; Department of Chemical and Biomolecular Engineering, Vanderbilt University, Nashville, TN, 37240, United States; Donald Danforth Plant Science Center, Olviette, MO, 63132, United States; Donald Danforth Plant Science Center, Olviette, MO, 63132, United States; Donald Danforth Plant Science Center, Olviette, MO, 63132, United States; United States Department of Agriculture, Agricultural Research Service, Washington, DC, 20250, United States; Department of Chemical Engineering, Massachusetts Institute of Technology, Cambridge, MA, 02139, United States; Department of Chemical and Biomolecular Engineering, Vanderbilt University, Nashville, TN, 37240, United States; Department of Molecular Physiology and Biophysics, Vanderbilt University, Nashville, TN, 37240, United States

## Abstract

**Summary:**

The analysis of stable isotope labeling experiments requires accurate, efficient, and reproducible quantification of mass isotopomer distributions (MIDs), which is not a core feature of general-purpose metabolomics software tools that are optimized to quantify metabolite abundance. Here, we present PIRAMID (Program for Integration and Rapid Analysis of Mass Isotopomer Distributions), a MATLAB-based tool that addresses this need by offering a user-friendly, graphical user interface-driven program to automate the extraction of isotopic information from mass spectrometry (MS) datasets. This tool can simultaneously extract ion chromatograms for various metabolites from multiple data files in common vendor–agnostic file formats, locate chromatographic peaks based on a targeted list of characteristic ions and retention times, and integrate MIDs for each target ion. These MIDs can be corrected for natural isotopic background based on the user-defined molecular formula of each ion. PIRAMID offers support for datasets acquired from low- or high-resolution MS, and single (MS) or tandem (MS/MS) instruments. It also enables the analysis of single or dual labeling experiments using a variety of isotopes (i.e. ^2^H, ^13^C, ^15^N, ^18^O, ^34^S).

**Data availability and implementation:**

MATLAB p-code files are freely available for non-commercial use and can be downloaded from https://mfa.vueinnovations.com/. Commercial licenses are also available. All the data presented in this publication are available under the “Help_menu” folder of the PIRAMID software.

## Graphical Abstract

**Figure:**
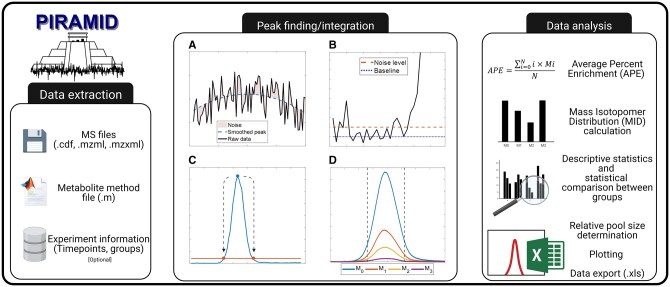

## 1 Introduction

Rapid advances in mass spectrometry (MS) technologies have propelled the broad usage of stable isotopes in biological “omics” studies (Han *et al.* 2008, Lehmann 2017). However, the lack of efficient and reproducible data analysis workflows remains a major bottleneck for many omics experiments (Cappadona *et al.* 2012). Various software tools have been developed to automate the processing of MS datasets (MacLean *et al.* 2010, Scheltema *et al.* 2011, Clasquin *et al.* 2012, Poskar *et al.* 2012, Chokkathukalam *et al.* 2013, Kiefer *et al.* 2013, Wills *et al.* 2017, Dagley and McConville 2018, Ji *et al.* 2018, Agrawal *et al.* 2019), but support for high-throughput analysis of stable isotope labeling experiments is limited. Specifically, isotope enrichments computed from raw MS data are sensitive to parameter values and require robust algorithms to minimize bias in the results (Antoniewicz *et al.* 2007, Krämer *et al.* 2018). Furthermore, existing tools perform only a subset of steps and may not be applicable to certain types of data (e.g. MS/MS, high-resolution MS, multi-isotope labeling experiments, etc.). To our knowledge, there is no publicly available software package that (i) has been optimized to accurately extract the mass isotopomer distributions (MIDs) of multiple target metabolites from a batch of metabolomics data files, (ii) automates the entire analysis workflow from start to finish, and (iii) supports a broad range of MS instruments and metabolomics experimental designs. (See the Glossary in Supplementary Section S4 for definitions of relevant terms)

In this article, we introduce PIRAMID (Program for Integration and Rapid Analysis of Mass Isotopomer Distributions): a vendor–agnostic tool optimized to analyze data from targeted metabolomics experiments involving stable isotopes. By automating the process of extracting, integrating, and analyzing high-throughput MS data in a user-friendly environment, we expect PIRAMID will enable a growing number of researchers to incorporate stable isotopes into their metabolomics studies.

## 2 Materials and methods

The workflow of PIRAMID can be broken down into three steps: (i) data extraction, (ii) peak finding and integration, and (iii) data analysis and output. A brief description of each step is provided below. The supported MS data types and a detailed explanation of the algorithms implemented in PIRAMID can be found in the Supplementary Note.

### 2.1 Data extraction

PIRAMID requires two file types: MS data files in .cdf, .mzml, or .mzxml formats and a MATLAB .m method file containing information describing the target compounds [e.g. their retention times (RTs), characteristic ion(s), and isotopologues to be quantified]. Plugins are integrated into the main graphical user interface to automate the creation and updating of the method file.

### 2.2 Peak finding and integration

For each of the target compounds in the method file, a peak is matched and assigned using a probabilistic method based on the compound’s characteristic ion(s) and expected RT provided in the method file. A composite peak is then created for each isotopic cluster, which contains all the isotopologues associated with a user-specified target ion of interest, by summing the intensities of the isotopologues to be quantified. The extracted ion chromatograms (EICs) of the composite peak and each individual isotopologue are smoothed using a Savitzky–Golay filter, the noise level is estimated, and the baseline of each EIC is calculated.

Integration bounds for quantifying each isotopologue are determined by scanning away from the apex of the composite peak in both directions until the signal intensity drops below a threshold that depends on the baseline and average noise level. Asymmetric peaks are corrected by adjusting the distance between the apex and the farthest edge on either side. The same integration bounds are applied to quantify all isotopologues within a given isotopic cluster, which ensures accurate quantification of low abundance isotopologues that may exhibit poor peak shape (Antoniewicz *et al.* 2007).

The integration of each EIC is computed as the sum of intensities between the peak edges, and the MID of each target ion is calculated as the relative area abundance of its isotopologues. The MIDs can be corrected for natural isotope abundance using different algorithms based on the type of MS data provided.

### 2.3 Data output

The output of the program is exportable as a .xls file containing the integrated MIDs, average percent enrichments (APEs), and ion abundances of target metabolites detected in each MS data file. If additional information on the corresponding sample time points and experimental groups is specified by the user, an additional sheet will include simple descriptive statistics (mean and standard deviation) for each combination of time point and experimental group, the root-mean-square error between the MIDs of unlabeled samples and their theoretical values, and the results of any requested statistical comparisons.

## 3 Results

For comparison purposes, two datasets were analyzed using PIRAMID, El-MAVEN (Agrawal *et al.* 2019), and Skyline (MacLean *et al.* 2010). The first dataset was acquired from a mixture of 20 unlabeled metabolites—including amino acids, sugars, and TCA cycle intermediates at three different concentrations (62.5, 125, and 250 µM)—using a SeQuant ZIC-HILIC column coupled to a Thermo Q-Exactive Mass Spectrometer in both positive and negative ionization modes, following the chromatographic conditions previously described (Kambhampati *et al.* 2021). The second dataset was acquired from a series of glutamine standards—unlabeled, [1-^13^C], [1,2-^13^C_2_], [^15^N_2_], and [U-^13^C_5_] (Cambridge Isotope Laboratories) at a constant concentration of 10 µM—using a Waters XBridge Amide HILIC column coupled to a Thermo Q-Exactive Mass Spectrometer in positive ion mode. Amino acids were analyzed by monitoring all combinations of carbon and nitrogen isotopologues, whereas the remaining metabolites were analyzed by monitoring only the carbon isotopologues.

The accuracy of each software tool was assessed as the error between the theoretically predicted MIDs and the empirically determined MIDs for the metabolites analyzed (Supplementary Section S2). In all cases, PIRAMID provided MIDs that were at least as accurate as other publicly available tools that were tested (Supplementary Section S3) while automating many tedious steps involved in the data processing workflow. Furthermore, PIRAMID provides several advanced features that are not available in other commonly used programs that have been previously assessed (Weber *et al.* 2017, Capellades *et al.* 2021, Rampler *et al.* 2021) (Table 1).

**Table 1.** Feature comparison across multiple freely available programs used to analyze metabolomics experiments involving stable isotopes.^a^

Features	iMS2Flux	eMZed3	TarMet	DExSI	AssayR	mzMatch-ISO	El-MAVEN	Skyline	PIRAMID
Load .cdf files			✔	✔					✔
Load .mzml/.mzxml files		✔	✔		✔		✔	✔	✔
Warp retention times		✔	✔						✔
Supports ^2^H	✔		✔	✔	✔		✔	✔	✔
Supports ^13^C	✔		✔	✔	✔	✔	✔	✔	✔
Supports ^15^N	✔		✔	✔	✔		✔	✔	✔
Supports ^18^O	✔		✔						✔
Supports ^34^S	✔						✔		✔
Supports MS/MS		✔	✔			✔	✔	✔	✔
Supports high-resolution MS	✔	✔	✔		✔	✔	✔	✔	✔
Supports dual-label experiments		✔						✔	✔
Consistent integration bounds for isotopic clusters									✔
MID calculation	✔	✔	✔	✔	✔				✔
Percent enrichment calculation				✔					✔
Natural abundance correction	✔			✔					✔
File/group comparisons		✔		✔		✔	✔	✔	✔
Visualize MIDs in real time before exporting		✔	✔	✔	✔	✔			✔
Graphical user interface-based			✔	✔			✔	✔	✔

a PIRAMID offers a wide range of capabilities focused on simplifying the data analysis pipeline. The use of fixed integration bounds for all the isotopologues within an isotopic cluster improves the accuracy of MID quantification. The ability to correct for natural isotope abundance in experiments involving dual-labeled tracers or MS/MS acquisition provides added versatility in processing stable isotope data.

## 4 Conclusions

PIRAMID is a user-friendly tool that automates the extraction, quantification, and analysis of MS datasets obtained from stable isotope labeling experiments. PIRAMID can be used for many purposes including: (i) automatic peak finding and integration of MIDs of multiple target metabolites from a batch of MS data files; (ii) quantification and normalization of integrated ion counts of the target metabolites; (iii) generation of statistical comparisons and plots to identify significant differences between two or more experimental conditions or sample time points; and (iv) exporting of data into .xls format that can be readily imported by spreadsheet programs or other tools. PIRAMID is expected to accelerate the analysis of high-throughput omics experiments, thus eliminating one of the major bottlenecks in the field.

## Supplementary Material

btad661_Supplementary_Data
