# Supplementary material for: Program for Integration and Rapid Analysis of Mass Isotopomer Distributions (PIRAMID)
Source: Bioinformatics. 2023 Oct 27;39(11):btad661. doi: 10.1093/bioinformatics/btad661 (PMC10636274; doi:10.1093/bioinformatics/btad661)
Supplement: btad661_Supplementary_Data [file btad661_supplementary_data.zip › PIRAMID_-_App_note_Supp_V5_clean.docx]

**Title: Program for Integration and Rapid Analysis of Mass Isotopomer Distributions (PIRAMID)**

**Authors:** Javier D. Gomez^1^, Martha L. Wall^1,†^, Mohsin Rahim^1^, Shrikaar Kamphampati^2^, Bradley S. Evans^2^, Doug K. Allen^2,3^, Maciek R. Antoniewicz^4,‡^, Jamey D. Young^1,5,*^.

^1^ Department of Chemical and Biomolecular Engineering, Vanderbilt University, USA
^2^ Donald Danforth Plant Science Center, USA
^3^ United States Department of Agriculture, Agriculture Research Service, USA
^4^ Department of Chemical Engineering, Massachusetts Institute of Technology, USA
^5^ Department of Molecular Physiology and Biophysics, Vanderbilt University, USA

^†^ Current affiliation: AbbVie, USA
^‡^ Current affiliation: Department of Chemical Engineering, University of Michigan, USA

^*^ To whom correspondence should be addressed:
Jamey D. Young
PMB 351604
Nashville, TN, 37064, USA
j.d.young@vanderbilt.edu

This document contains the following information in support of the primary article.

Supplementary note. Detailed explanation of the implemented algorithms.

Section S-1. List of standards used in the test cases.

Section S-2. Uncorrected MIDs of standards determined using El-MAVEN, Skyline, and PIRAMID.

Section S-3. Average errors in MIDs determined from each program.

Section S-4. Glossary of used terminology.

**Supplementary note.** Detailed explanation of the implemented algorithms.

*Data loading*

PIRAMID requires two file types: MS data files and a Matlab .m method file containing information describing the target compounds (e.g., their retention times (RTs), characteristic ion(s), and isotopologues to be quantified). Plugins are integrated into the main GUI to automate the creation and updating of the method file. Dual-labeled metabolites are entered by specifying the combinations of labeled atoms sorted by the first tracer atom, which is assumed to be carbon, followed by the second tracer atom (e.g., Glu-C0N0, Glu-C1N0, Glu-C2N0, Glu-C0N1, Glu-C1N1, Glu-C2N1…).

The MS data needs to be converted to the following vendor-agnostic formats: .cdf, .mzml, or .mzxml. MS1- and MS2-level data are accepted, regardless of the ionization mode. The following modes of operation of MS data are compatible: Selected Ion Monitoring, Product Ion Scan, Precursor Ion Scan, Selected Reaction Monitoring, Multiple Reaction Monitoring, Full Scan MS, Data Dependent Scan, Data Independent Scan.

Upon loading the files, the raw EICs for each target compound are extracted and loaded into a Matlab structure containing the RT and intensity information of the monitored ions within a time window surrounding the expected RT. If multiple signals match the same *m/z* range (to within the *m/z* tolerance specified by the user), a composite EIC is created by summing the corresponding signals.

*Data preprocessing*

In the specific case where RT drift occurs across multiple files, the EICs can be aligned using a method based on the built-in Matlab functions “*alignsignals*” and “*delayseq*”.

*Peak finding*

For each of the target compounds in the method file, a peak is matched and assigned using a probabilistic method based on the characteristic ion(s) and expected RT provided in the method file. First, the total intensity across all the characteristic ions of the target metabolite is summed and multiplied by a probability function that accounts for the proximity of the peak apex to its expected RT. If multiple characteristic ions are specified, a second probability function is multiplied that accounts for their expected relative intensities. The most likely peak location is defined as the RT value where the product of the probabilities is the greatest. The apex of the peak is found using the Matlab built-in function “*findpeaks*”.

*Peak assignment and smoothing*

A composite peak is created for each isotopic cluster by summing the EICs of the isotopologues to be quantified. Next, the EICs of the composite peak and each individual isotopologue are smoothed using a Savitzky-Golay filter of user-specified order polynomial (second order is the default).

*Noise level determination*

The noise level is estimated as the mean difference between the raw and smoothed signals.

*Baseline calculation*

The baseline for each EIC is determined using the second derivative of the intensity with respect to time. From the apex, the algorithm scans in both directions until it finds five consecutive measurements where the second derivative of the intensity with respect to time has a value below a user-defined value (0.3 [counts/min^2^] by default). If this process is not successful, the baseline is defined by finding the average intensity of all the points that fall below the noise level. This is achieved by scanning each EIC from the apex in both directions until five consecutive measurements have intensities below the aforementioned threshold. If no points are found, the noise level is doubled, and the process is repeated.

*Peak edge determination*

Integration bounds for quantifying each isotopologue are determined by scanning away from the apex of the composite peak in both directions until the signal intensity drops below a threshold level (defined as the sum of the baseline and the average noise level, multiplied by a baseline factor [1 by default]). To avoid pitfalls arising from common artifacts like peak tailing and shouldering, the edges of asymmetric peaks (defined as peaks with significantly different distances between the apex and the upper and lower integration bounds) are corrected by reducing the distance between the apex and the farthest edge on either side so it matches the distance between the apex and the nearest edge.

*Integration and MID determination*

The integration of each EIC is computed as the sum of intensities between the peak edges, and the MID of each target ion is calculated as the relative area abundance of its isotopologues. The same integration bounds are applied to quantify all isotopologues within a given isotopic cluster, which ensures accurate quantification of low abundance isotopologues that may exhibit poor peak shape (Antoniewicz *et al.*, 2007).

*Correction for natural abundance*

The MIDs can be corrected for natural isotope abundance based on the chemical formulae of the ions using different methods depending on the type of MS data provided. If low-resolution data are used, a previously described correction matrix is applied (Fernandez *et al.*, 1996). If tandem MS/MS data are used, a different correction matrix is applied that requires the formulae of both precursor and product ions (Choi and Antoniewicz, 2019). Finally, if high-resolution data are used in conjunction with a dual-labeled tracer, the data are corrected for the contribution of labeled atoms using an approach similar to the one used in the software package Accucor2 (Wang *et al.*, 2021), without accounting for the non-tracer elements.

*Post-processing calculations*

Using the corrected data, PIRAMID calculates the average percent enrichment (APE) of each target ion, following Equation 1, where $M_{i}$ represents the relative abundance of the isotopologue with *i* heavy atoms, and $N$ represents the maximum number of atoms than can be enriched by the isotope tracer. If time point information is available, the program will calculate the root mean-square error between the empirically determined MIDs of unlabeled samples (t=0) and their theoretical values computed based on the chemical formula of each target ion and the natural abundance of stable isotopes for each element included in the formula.

$\frac{\sum_{i=0}^{N} i\times Mi}{N}$ (1)

Optionally, a statistical analysis of up to 10 combinations of sample time points and experimental groups can be performed. Once the comparisons are specified, a two-tailed t-test is performed to compare the APEs of selected target ions and the intensities of each constituent isotopologue. The user can also choose to normalize the total ion counts of target ions to an internal standard peak in order to compare relative metabolite abundances across different samples.

**Section S-1.** List of standards used in the test cases.

**Table S-1** List of metabolites used in the test comparisons and their corresponding method parameters. The mixture is composed of 12 amino acids, 4 TCA cycle intermediates, and 4 sugars. Several glutamine standards were used with different isotopic compositions: unlabeled, [1-^13^C], [1,2-^13^C_2_], [^15^N_2_], and [U-^13^C_5_]. All ^13^C and ^15^N isotopologues were monitored up to the maximum enrichment indicated in the rightmost column.

| **Metabolite** | **Retention Time** | **Formula** | **Monitored isotopes** |
| --- | --- | --- | --- |
| Alanine | 7.75 | C3H7NO2 | C3N1 |
| Aspartate | 7.75 | C4H7NO4 | C4N1 |
| Glutamate | 7.73 | C5H9NO4 | C5N1 |
| Isoleucine | 6.10 | C6H13NO2 | C6N1 |
| Leucine | 6.37 | C6H13NO2 | C6N1 |
| Methionine | 6.14 | C5H11NO2S | C5N1 |
| Phenylalanine | 5.25 | C9H11NO2 | C9N1 |
| Proline | 7.50 | C5H9NO2 | C5N1 |
| Serine | 8.05 | C3H7NO3 | C3N1 |
| Threonine | 7.45 | C4H9NO3 | C4N1 |
| Tyrosine | 6.95 | C9H11NO3 | C9N1 |
| Valine | 6.90 | C5H11NO2 | C5N1 |
| Fumarate | 7.35 | C4H4O4 | C4 |
| Succinate | 7.25 | C4H6O4 | C4 |
| Malate | 7.37 | C4H6O5 | C4 |
| Citrate | 6.20 | C6H8O7 | C6 |
| Glucose | 9.10 | C6H12O6 | C6 |
| Fructose | 7.77 | C6H12O6 | C6 |
| Glucose-1-Phosphate | 8.25 | C6H13O9P | C6 |
| Glucose-6-Phosphate | 8.50 | C6H13O9P | C6 |
| Glutamine | 4.90 | C5H10N2O3 | C5N2 |

**Section S-2.** Uncorrected MIDs of standards determined using El-MAVEN, Skyline, and PIRAMID.

**Table S-2.1.** Integrated MIDs of metabolite mixture using different programs. Given the large size of the data set, the results are provided in a separate .xls file.

**Table S-2.2.** Integrated MIDs of glutamine standards using different programs. The expected MIDs used for error calculations are shown in the last table.

|  | **El-MAVEN** | | | | |
| --- | --- | --- | --- | --- | --- |
|  | **Unlabeled** | **[1-^13^C]** | **[^15^N_2_]** | **[1,2-^13^C_2_]** | **[U-^13^C_5_]** |
| **C0N0** | 93.90% | 0.53% | 0.05% | 0.07% | 0.06% |
| **C1N0** | 5.26% | 93.86% | 0.02% | 1.37% | 0.00% |
| **C2N0** | 0.12% | 4.13% | 0.01% | 94.37% | 0.06% |
| **C3N0** | 0.01% | 0.15% | 0.00% | 3.21% | 0.21% |
| **C4N0** | 0.00% | 0.01% | 0.00% | 0.13% | 3.96% |
| **C5N0** | 0.00% | 0.57% | 0.00% | 0.00% | 95.51% |
| **C0N1** | 0.63% | 0.01% | 0.50% | 0.00% | 0.01% |
| **C1N1** | 0.02% | 0.68% | 0.00% | 0.07% | 0.00% |
| **C2N1** | 0.00% | 0.02% | 0.00% | 0.76% | 0.00% |
| **C3N1** | 0.00% | 0.00% | 0.00% | 0.02% | 0.00% |
| **C4N1** | 0.00% | 0.00% | 0.00% | 0.00% | 0.15% |
| **C5N1** | 0.00% | 0.00% | 0.00% | 0.00% | 0.00% |
| **C0N2** | 0.02% | 0.01% | 93.98% | 0.01% | 0.02% |
| **C1N2** | 0.00% | 0.00% | 5.36% | 0.00% | 0.00% |
| **C2N2** | 0.02% | 0.01% | 0.07% | 0.00% | 0.03% |
| **C3N2** | 0.00% | 0.00% | 0.00% | 0.00% | 0.00% |
| **C4N2** | 0.02% | 0.00% | 0.00% | 0.00% | 0.00% |
| **C5N2** | 0.00% | 0.00% | 0.00% | 0.00% | 0.00% |

|  | **Skyline** | | | | |
| --- | --- | --- | --- | --- | --- |
|  | **Unlabeled** | **[1-^13^C]** | **[^15^N_2_]** | **[1,2-^13^C_2_]** | **[U-^13^C_5_]** |
| **C0N0** | 93.13% | 0.58% | 0.07% | 0.07% | 0.07% |
| **C1N0** | 5.34% | 91.96% | 0.02% | 1.33% | 0.00% |
| **C2N0** | 0.11% | 4.23% | 0.09% | 94.52% | 0.09% |
| **C3N0** | 0.00% | 0.04% | 0.00% | 3.04% | 0.20% |
| **C4N0** | 0.00% | 0.00% | 0.00% | 0.02% | 3.64% |
| **C5N0** | 0.00% | 0.55% | 0.00% | 0.00% | 95.08% |
| **C0N1** | 0.63% | 0.03% | 0.45% | 0.02% | 0.01% |
| **C1N1** | 0.02% | 0.73% | 0.00% | 0.01% | 0.00% |
| **C2N1** | 0.00% | 0.03% | 0.00% | 0.77% | 0.00% |
| **C3N1** | 0.00% | 0.00% | 0.01% | 0.03% | 0.02% |
| **C4N1** | 0.00% | 0.00% | 0.00% | 0.00% | 0.07% |
| **C5N1** | 0.71% | 1.81% | 0.00% | 0.16% | 0.44% |
| **C0N2** | 0.01% | 0.00% | 94.49% | 0.00% | 0.03% |
| **C1N2** | 0.00% | 0.00% | 4.72% | 0.00% | 0.00% |
| **C2N2** | 0.01% | 0.00% | 0.04% | 0.01% | 0.01% |
| **C3N2** | 0.01% | 0.01% | 0.02% | 0.02% | 0.26% |
| **C4N2** | 0.01% | 0.00% | 0.00% | 0.00% | 0.00% |
| **C5N2** | 0.01% | 0.02% | 0.08% | 0.01% | 0.06% |

|  | **PIRAMID** | | | | |
| --- | --- | --- | --- | --- | --- |
|  | **Unlabeled** | **[1-^13^C]** | **[^15^N_2_]** | **[1,2-^13^C_2_]** | **[U-^13^C_5_]** |
| **C0N0** | 93.96% | 0.50% | 0.04% | 0.07% | 0.06% |
| **C1N0** | 5.26% | 93.94% | 0.03% | 1.36% | 0.00% |
| **C2N0** | 0.12% | 4.40% | 0.00% | 94.17% | 0.09% |
| **C3N0** | 0.00% | 0.05% | 0.00% | 3.20% | 0.20% |
| **C4N0** | 0.00% | 0.00% | 0.00% | 0.05% | 3.89% |
| **C5N0** | 0.00% | 0.00% | 0.00% | 0.00% | 94.49% |
| **C0N1** | 0.61% | 0.27% | 0.50% | 0.00% | 0.03% |
| **C1N1** | 0.03% | 0.69% | 0.00% | 0.14% | 0.00% |
| **C2N1** | 0.00% | 0.04% | 0.00% | 0.76% | 0.00% |
| **C3N1** | 0.00% | 0.00% | 0.00% | 0.04% | 0.06% |
| **C4N1** | 0.00% | 0.00% | 0.00% | 0.00% | 0.26% |
| **C5N1** | 0.00% | 0.00% | 0.09% | 0.19% | 0.69% |
| **C0N2** | 0.00% | 0.00% | 93.90% | 0.01% | 0.04% |
| **C1N2** | 0.00% | 0.00% | 5.31% | 0.00% | 0.00% |
| **C2N2** | 0.00% | 0.00% | 0.04% | 0.00% | 0.04% |
| **C3N2** | 0.00% | 0.10% | 0.01% | 0.00% | 0.08% |
| **C4N2** | 0.02% | 0.00% | 0.00% | 0.00% | 0.00% |
| **C5N2** | 0.00% | 0.00% | 0.08% | 0.00% | 0.06% |

|  | **Isotopologue Abundance** | | | | |
| --- | --- | --- | --- | --- | --- |
|  | **Unlabeled** | **[1-^13^C]** | **[^15^N_2_]** | **[1,2-^13^C_2_]** | **[U-^13^C_5_]** |
| **C0N0** | 94.02% | 0.94% | 0.01% | 0.01% | 0.00% |
| **C1N0** | 5.14% | 94.14% | 0.00% | 1.88% | 0.00% |
| **C2N0** | 0.11% | 4.12% | 0.00% | 94.25% | 0.00% |
| **C3N0** | 0.00% | 0.07% | 0.00% | 3.09% | 0.09% |
| **C4N0** | 0.00% | 0.00% | 0.00% | 0.03% | 4.72% |
| **C5N0** | 0.00% | 0.00% | 0.00% | 0.00% | 94.46% |
| **C0N1** | 0.69% | 0.01% | 1.87% | 0.00% | 0.00% |
| **C1N1** | 0.04% | 0.69% | 0.10% | 0.01% | 0.00% |
| **C2N1** | 0.00% | 0.03% | 0.00% | 0.69% | 0.00% |
| **C3N1** | 0.00% | 0.00% | 0.00% | 0.02% | 0.00% |
| **C4N1** | 0.00% | 0.00% | 0.00% | 0.00% | 0.04% |
| **C5N1** | 0.00% | 0.00% | 0.00% | 0.00% | 0.70% |
| **C0N2** | 0.00% | 0.00% | 92.83% | 0.00% | 0.00% |
| **C1N2** | 0.00% | 0.00% | 5.08% | 0.00% | 0.00% |
| **C2N2** | 0.00% | 0.00% | 0.11% | 0.00% | 0.00% |
| **C3N2** | 0.00% | 0.00% | 0.00% | 0.00% | 0.00% |
| **C4N2** | 0.00% | 0.00% | 0.00% | 0.00% | 0.00% |
| **C5N2** | 0.00% | 0.00% | 0.00% | 0.00% | 0.00% |

**Section S-3.** Average errors in MIDs determined from each program.

**Table S-3.1.** Calculated errors for each program based on the difference between the expected MIDs and the measured MIDs for the metabolite mixture.

|  | **El-MAVEN** | | | **Skyline** | | | **PIRAMID** | | |
| --- | --- | --- | --- | --- | --- | --- | --- | --- | --- |
|  | **62.5 µM** | **125 µM** | **250 µM** | **62.5 µM** | **125 µM** | **250 µM** | **62.5 µM** | **125 µM** | **250 µM** |
| **Ala** | 0.168% | 0.247% | 0.100% | 0.321% | 0.267% | 0.343% | 0.256% | 0.216% | 0.273% |
| **Asp** | 0.051% | 0.068% | 0.047% | 0.114% | 0.047% | 0.270% | 0.049% | 0.025% | 0.084% |
| **Cit** | 0.858% | 0.460% | 0.474% | 0.510% | 0.452% | 1.866% | 0.483% | 0.496% | 0.999% |
| **Fru** | 0.590% | 0.549% | 0.403% | 0.706% | 3.435% | 2.312% | 0.706% | 0.364% | 0.967% |
| **Fum** | 0.618% | 0.274% | 0.313% | 0.665% | 0.443% | 0.355% | 0.354% | 0.366% | 0.392% |
| **G1P** | 0.097% | 0.089% | 0.162% | 0.161% | 0.148% | 0.183% | 0.116% | 0.165% | 0.140% |
| **G6P** | 0.091% | 0.085% | 0.157% | 0.104% | 0.108% | 0.098% | 0.039% | 0.099% | 0.048% |
| **Glc** | 0.445% | 0.660% | 0.401% | 0.841% | 0.760% | 0.907% | 0.799% | 0.477% | 0.885% |
| **Glu** | 0.043% | 0.066% | 0.170% | 0.654% | 0.111% | 0.618% | 0.105% | 0.024% | 0.040% |
| **Iso** | 0.009% | 0.020% | 0.053% | 0.111% | 0.181% | 0.063% | 0.022% | 0.044% | 0.016% |
| **Leu** | 0.009% | 0.020% | 0.053% | 0.149% | 0.135% | 0.105% | 0.028% | 0.069% | 0.017% |
| **Mal** | 0.301% | 0.334% | 0.294% | 0.438% | 0.385% | 0.442% | 0.282% | 0.273% | 0.272% |
| **Met** | 0.071% | 0.105% | 0.045% | 0.241% | 0.202% | 0.651% | 0.147% | 0.057% | 0.211% |
| **Phe** | 0.024% | 0.023% | 0.021% | 0.115% | 0.107% | 0.117% | 0.026% | 0.020% | 0.023% |
| **Pro** | 0.073% | 0.070% | 0.144% | 0.133% | 0.138% | 0.116% | 0.044% | 0.085% | 0.062% |
| **Ser** | 9.439% | 6.365% | 2.664% | 16.260% | 3.704% | 18.488% | 2.743% | 5.128% | 0.459% |
| **Suc** | 0.263% | 0.305% | 0.251% | 0.614% | 0.637% | 0.454% | 0.324% | 0.309% | 0.442% |
| **Thr** | 0.396% | 0.342% | 0.107% | 3.254% | 3.359% | 2.358% | 0.959% | 0.306% | 0.692% |
| **Tyr** | 0.024% | 0.019% | 0.022% | 0.103% | 0.117% | 0.081% | 0.014% | 0.013% | 0.035% |
| **Val** | 0.060% | 0.050% | 0.013% | 0.085% | 0.124% | 0.153% | 0.023% | 0.025% | 0.069% |
| **Avg-Measured** | 0.681% | 0.508% | 0.295% | 1.279% | 0.743% | 1.499% | 0.376% | 0.428% | 0.306% |
| **Avg Program** | 0.495% | | | 1.174% | | | 0.370% | | |

**Figure S.3.1.** Average errors across each concentration tested. The errors are calculated as the average of the differences between the measured and expected MIDs. Only metabolites that showed errors below 1% are shown.

**Figure S.3.2.** Average errors across each concentration tested. The errors are calculated as the average of the differences between the measured and expected MIDs. Only metabolites that showed errors above 1% are shown.

Higher errors in the serine measurement can be explained due to an interfering peak in the M+2 ^13^C isotopologue that biases the results (Fig S.3.3). PIRAMID is capable of adjusting the baseline to reduce the error. Similar defects were observed in other metabolite peaks such as fructose and threonine. Properly accounting for and correcting these defects is crucial to ensure accurate quantification of the MIDs.

**
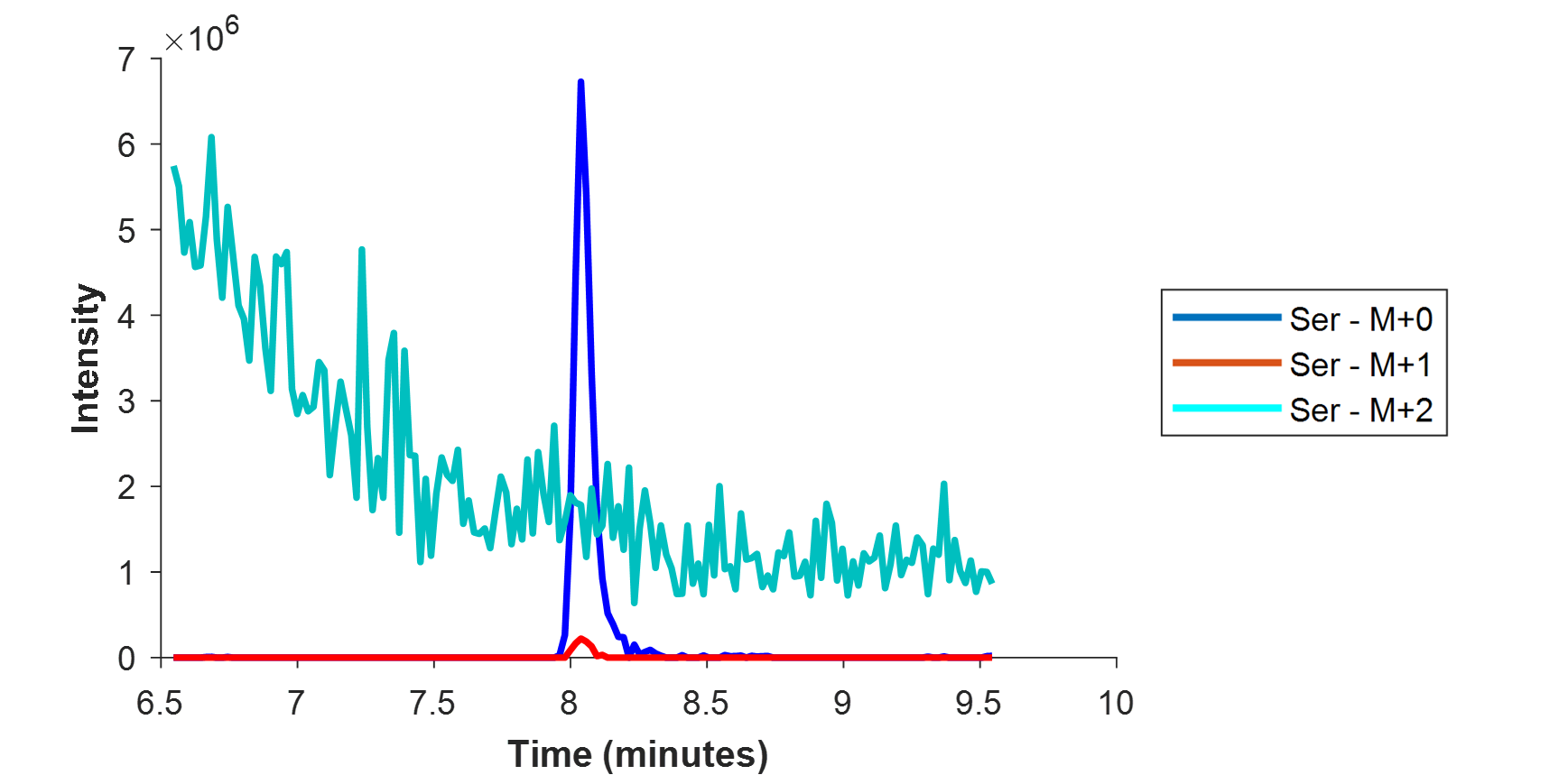
Figure S.3.3.** Serine ^13^C isotopologues. The shoulder of an interfering ion with the same mass as the M+2 ^13^C isotopologue leads to overestimation of its peak area and skews the results. PIRAMID is capable of adjusting the high baseline and estimates the MID more accurately than the other programs tested.

**Table S-3.2.** Calculated errors for each program based on the difference between the expected MIDs and the measured MIDs for each glutamine standard.

|  | **Maven** | | | | |
| --- | --- | --- | --- | --- | --- |
|  | **Unlabeled** | **[1-^13^C]** | **[1,2-^13^C_2_]** | **[^15^N_2_]** | **[U-^13^C_5_]** |
| **C0N0** | 0.139% | 0.442% | 0.057% | 0.044% | 0.065% |
| **C1N0** | 0.146% | 0.441% | 0.498% | 0.030% | 0.010% |
| **C2N0** | 0.040% | 0.129% | 0.030% | 0.060% | 0.073% |
| **C3N0** | 0.002% | 0.025% | 0.178% | 0.003% | 0.099% |
| **C4N0** | 0.003% | 0.010% | 0.013% | 0.002% | 0.671% |
| **C5N0** | 0.006% | 0.577% | 0.003% | 0.003% | 0.715% |
| **C0N1** | 0.058% | 0.165% | 0.017% | 1.333% | 0.015% |
| **C1N1** | 0.010% | 0.000% | 0.239% | 0.102% | 0.006% |
| **C2N1** | 0.001% | 0.011% | 0.024% | 0.002% | 0.001% |
| **C3N1** | 0.000% | 0.000% | 0.023% | 0.000% | 0.001% |
| **C4N1** | 0.001% | 0.002% | 0.001% | 0.001% | 0.364% |
| **C5N1** | 0.000% | 0.000% | 0.000% | 0.000% | 0.695% |
| **C0N2** | 0.008% | 0.011% | 0.019% | 1.020% | 0.016% |
| **C1N2** | 0.002% | 0.002% | 0.003% | 0.387% | 0.004% |
| **C2N2** | 0.000% | 0.000% | 0.001% | 0.111% | 0.000% |
| **C3N2** | 0.000% | 0.000% | 0.000% | 0.001% | 0.000% |
| **C4N2** | 0.000% | 0.000% | 0.000% | 0.000% | 0.000% |
| **C5N2** | 0.000% | 0.000% | 0.000% | 0.000% | 0.001% |
| **Avg-Measured** | 0.023% | 0.101% | 0.061% | 0.172% | 0.152% |
| **Avg Program** | 0.102% | | | | |
|  |  |  |  |  |  |

|  | **Skyline** | | | | |
| --- | --- | --- | --- | --- | --- |
|  | **Unlabeled** | **[1-^13^C]** | **[1,2-^13^C_2_]** | **[^15^N_2_]** | **[U-^13^C_5_]** |
| **C0N0** | 0.883% | 0.364% | 0.062% | 0.060% | 0.070% |
| **C1N0** | 0.198% | 2.189% | 0.556% | 0.018% | 0.005% |
| **C2N0** | 0.001% | 0.111% | 0.267% | 0.093% | 0.091% |
| **C3N0** | 0.001% | 0.029% | 0.047% | 0.000% | 0.106% |
| **C4N0** | 0.001% | 0.004% | 0.009% | 0.000% | 1.077% |
| **C5N0** | 0.001% | 0.550% | 0.000% | 0.001% | 0.625% |
| **C0N1** | 0.058% | 0.026% | 0.018% | 1.415% | 0.012% |
| **C1N1** | 0.018% | 0.042% | 0.001% | 0.102% | 0.001% |
| **C2N1** | 0.001% | 0.002% | 0.074% | 0.002% | 0.000% |
| **C3N1** | 0.002% | 0.003% | 0.008% | 0.012% | 0.018% |
| **C4N1** | 0.000% | 0.000% | 0.000% | 0.000% | 0.037% |
| **C5N1** | 0.712% | 1.815% | 0.155% | 0.000% | 0.258% |
| **C0N2** | 0.006% | 0.003% | 0.000% | 1.665% | 0.032% |
| **C1N2** | 0.000% | 0.001% | 0.000% | 0.356% | 0.000% |
| **C2N2** | 0.015% | 0.004% | 0.007% | 0.072% | 0.014% |
| **C3N2** | 0.007% | 0.008% | 0.016% | 0.017% | 0.264% |
| **C4N2** | 0.007% | 0.000% | 0.000% | 0.000% | 0.000% |
| **C5N2** | 0.013% | 0.015% | 0.007% | 0.080% | 0.060% |
| **Avg-Measured** | 0.107% | 0.287% | 0.068% | 0.216% | 0.148% |
| **Avg Program** | 0.165% | | | | |
|  |  |  |  |  |  |

|  | **PIRAMID** | | | | |
| --- | --- | --- | --- | --- | --- |
|  | **Unlabeled** | **[1-^13^C]** | **[^15^N_2_]** | **[1,2-^13^C_2_]** | **[U-^13^C_5_]** |
| **C0N0** | 0.055% | 0.440% | 0.031% | 0.061% | 0.060% |
| **C1N0** | 0.120% | 0.204% | 0.029% | 0.522% | 0.000% |
| **C2N0** | 0.008% | 0.284% | 0.000% | 0.083% | 0.089% |
| **C3N0** | 0.001% | 0.018% | 0.000% | 0.110% | 0.106% |
| **C4N0** | 0.000% | 0.000% | 0.000% | 0.016% | 0.828% |
| **C5N0** | 0.000% | 0.000% | 0.000% | 0.000% | 0.035% |
| **C0N1** | 0.081% | 0.263% | 1.368% | 0.000% | 0.030% |
| **C1N1** | 0.008% | 0.002% | 0.102% | 0.126% | 0.000% |
| **C2N1** | 0.001% | 0.010% | 0.002% | 0.067% | 0.000% |
| **C3N1** | 0.000% | 0.000% | 0.000% | 0.017% | 0.059% |
| **C4N1** | 0.000% | 0.000% | 0.000% | 0.000% | 0.225% |
| **C5N1** | 0.000% | 0.000% | 0.090% | 0.190% | 0.005% |
| **C0N2** | 0.001% | 0.000% | 1.070% | 0.010% | 0.040% |
| **C1N2** | 0.000% | 0.001% | 0.235% | 0.000% | 0.000% |
| **C2N2** | 0.000% | 0.000% | 0.071% | 0.001% | 0.040% |
| **C3N2** | 0.000% | 0.100% | 0.009% | 0.000% | 0.080% |
| **C4N2** | 0.020% | 0.000% | 0.000% | 0.000% | 0.000% |
| **C5N2** | 0.000% | 0.000% | 0.080% | 0.000% | 0.059% |
| **Avg-Measured** | 0.016% | 0.073% | 0.172% | 0.067% | 0.092% |
| **Avg Program** | 0.084% | | | | |


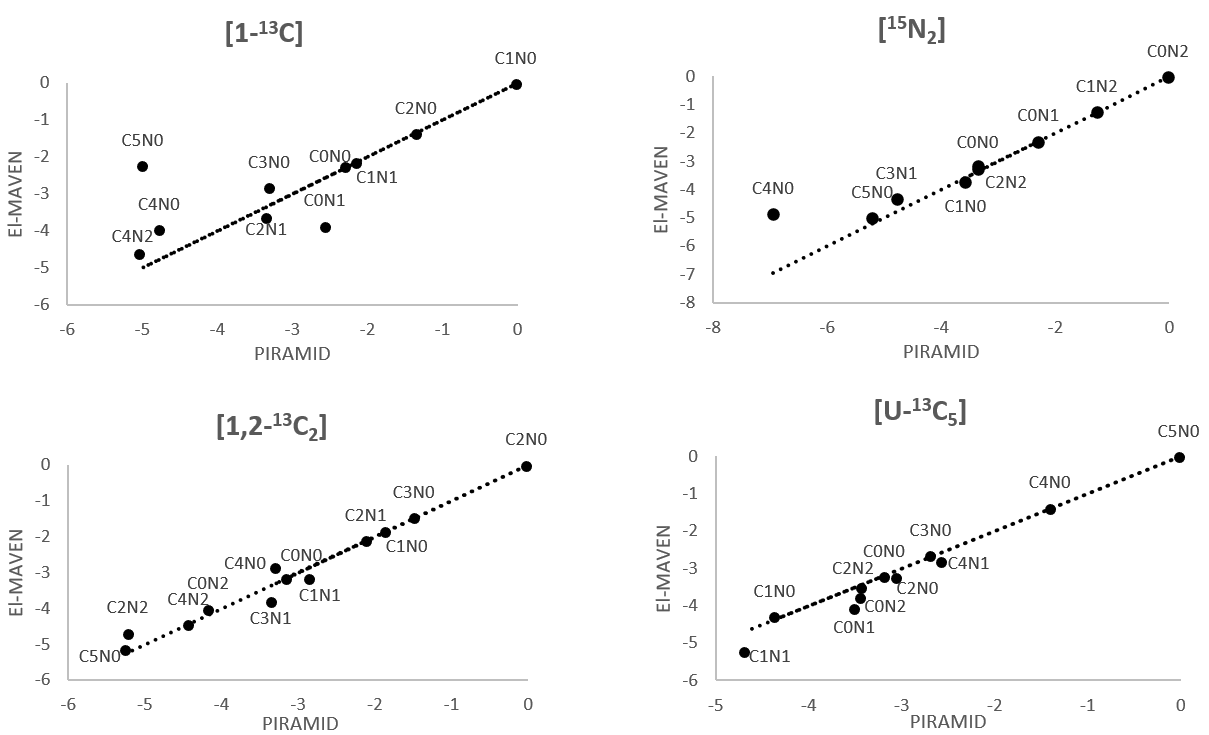

**Figure S.3.4.** Linearity analysis of the labeled standards analyzed using PIRAMID versus El-MAVEN. The axes labels indicate the relative abundance of each isotopologue on a log_10_ scale. Isotopologues that were not detected by any of the tools and showed a zero value in their intensity are not presented. The dotted line represents 1:1 agreement between the programs.


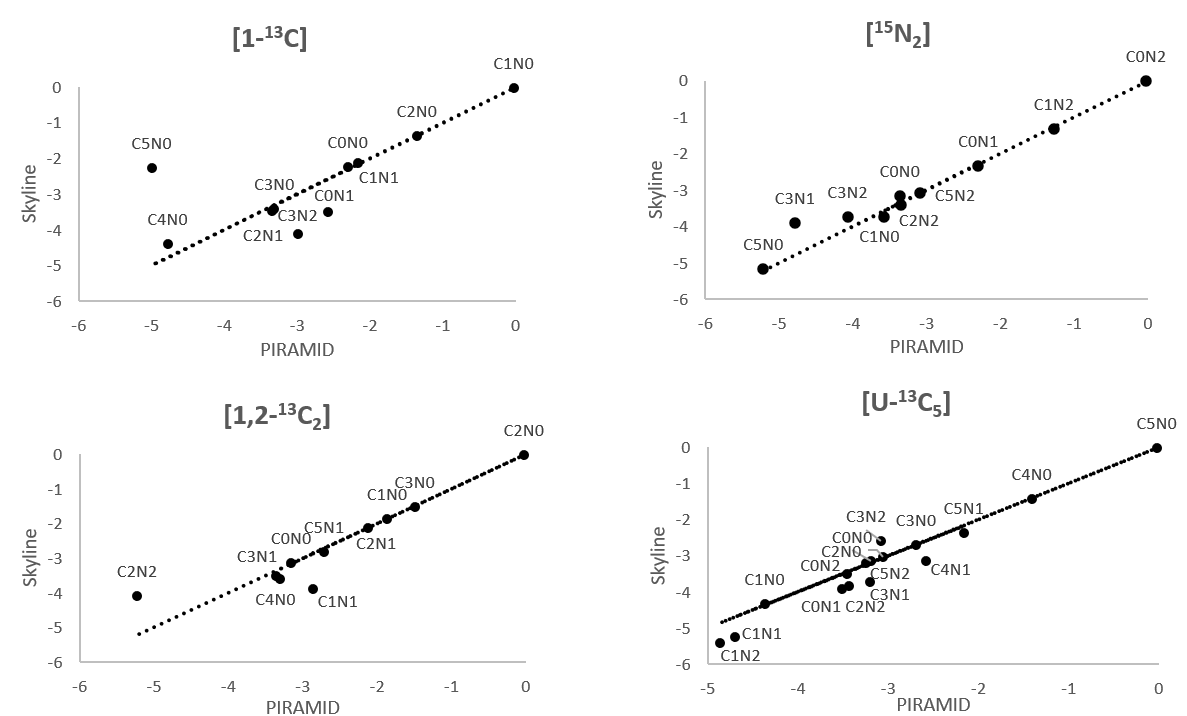

**Figure S.3.5.** Linearity analysis of the labeled standards analyzed using PIRAMID versus Skyline. The axes labels indicate the relative abundance of each isotopologue on a log_10_ scale. Isotopologues that were not detected by any of the tools and showed a zero value in their intensity are not presented. The dotted line represents 1:1 agreement between the programs.

A comparison of the performance of PIRAMID versus El-MAVEN and Skyline is presented in Figures S.3.4 and S.3.5 via a linearity analysis. The results show a high correlation for nearly all isotopologues. The measurement of the M+5 ^13^C isotopologue in the [1-^13^C]glutamine standard shows a deviation between PIRAMID and the other tools. In this case, PIRAMID matches the expected value precisely while the other tools overestimate the expected value. However, the errors are fall below <1% and are not considered significant. As all tools yield accurate results within the acceptable error threshold, no tool can be considered as more accurate than the others. Nonetheless, it can be concluded that PIRAMID is at least as accurate as the other publicly available tools tested.

**Section S-4.** Glossary of relevant terminology

**Isotopologue:** Isomers that differ only in their isotopic composition with otherwise identical structure, including isotopic substitutions of different elements (IUPAC, 2014). For example, [1-^13^C]glutamine, [^15^N_2_]glutamine, [1,2-^13^C_2_]glutamine, and [U-^13^C_5_]glutamine are isotopologues of glutamine.

**Mass Isotopomer:** Mass isotopomers are groups of isotopologues of the same mass that cannot be separated by mass spectrometry (Weindl *et al.*, 2015; Hellerstein and Neese, 1999). When measured at nominal mass resolution, mass isotopomers are denoted as *M+x*, where *x* is the nominal mass that is added to the monoisotopic mass.

**Isotopomer:** Isotopomers are isotopologues with the same isotopic substituents but in varying positions (Murray *et al.*, 2013). MS1 mass spectrometry is not able to resolve these isotopomers as they have the same exact mass. For example, [1-^13^C]glutamine and [2-^13^C]glutamine are isotopomers.

**Mass Isotopomer Distribution (MID)**: A data vector comprising the relative abundance of each mass isotopomer of a compound expressed as a fraction of the total measured pool. MID measurements are used to assess the level of enrichment produced by metabolomics experiments involving stable isotopes.

**References**

Antoniewicz,M.R. *et al.* (2007) Accurate assessment of amino acid mass isotopomer distributions for metabolic flux analysis. *Analytical Chemistry*, **79**, 7554–7559.

Choi,J. and Antoniewicz,M.R. (2019) Tandem mass spectrometry for 13C metabolic flux analysis: Methods and algorithms based on EMU framework. *Frontiers in Microbiology*, **10**.

Fernandez,C.A. *et al.* (1996) Correction of 13C mass isotopomer distributions for natural stable isotope abundance. *Journal of Mass Spectrometry*, **31**, 255–262.

Hellerstein,M.K. and Neese,R.A. (1999) Mass isotopomer distribution analysis at eight years: Theoretical, analytic, and experimental considerations. *Am J Physiol Endocrinol Metab*, **276**.

IUPAC (2014) Gold book - Compendium of chemical terminology Version 2.3.3.

Murray,K.K. *et al.* (2013) Definitions of terms relating to mass spectrometry (IUPAC Recommendations 2013). *Pure and Applied Chemistry*, **85**, 1515–1609.

Wang,Y. *et al.* (2021) AccuCor2: isotope natural abundance correction for dual-isotope tracer experiments. *Laboratory Investigation*, **101**, 1403–1410.

Weindl,D. *et al.* (2015) Non-targeted Tracer Fate Detection. *Methods Enzymol*, **561**, 277–302.
