# Supplementary material for: Program for Integration and Rapid Analysis of Mass Isotopomer Distributions (PIRAMID)
Source: Bioinformatics. 2023 Oct 27;39(11):btad661. doi: 10.1093/bioinformatics/btad661 (PMC10636274; doi:10.1093/bioinformatics/btad661)
Supplement: btad661_Supplementary_Data [file btad661_supplementary_data.zip › PIRAMID User Guide.pdf]

PIRAMID User Guide

Before running PIRAMID make sure that the Signal Processing Toolbox and the Statistics and Machine Learning Toolbox are installed.

In the help menu folder, there are two datasets that can be used to become familiar with the tool.

1. Glutamine labeling: Contains the files of a simulation of an increasing glutamine labeling with time in duplicate. The files come from a LCMS run.
2. Metabolite mixture: Contains the file of a mixture of unlabeled metabolite standards. The file comes from a GCMS run.

Table of contents

A. Extracting the MIDs from a raw file 2

B. Creating a new method file 5

C. Modifying an existing method. 7

E. Plotting the results 9

F. Normalize the enrichments to an internal standard. 10

G. Correcting for retention times drifts. 11

H. Adjusting advanced settings 12

A. Extracting the MIDs from a raw file.

1. Open Matlab 2018a or any more recent version.
2. Write PIRAMID in the Command Window

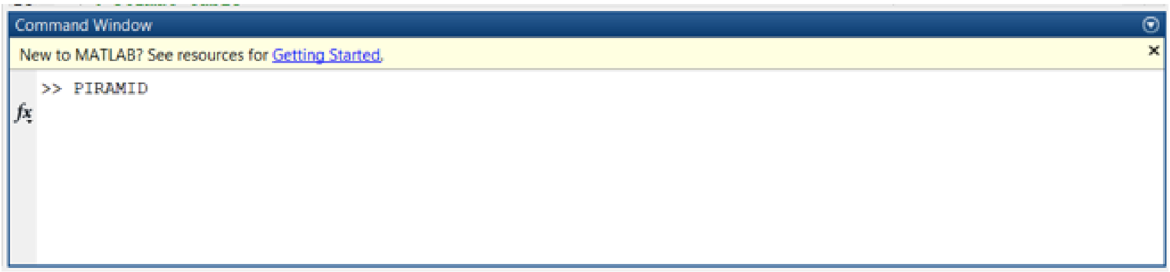

The following GUI should appear.

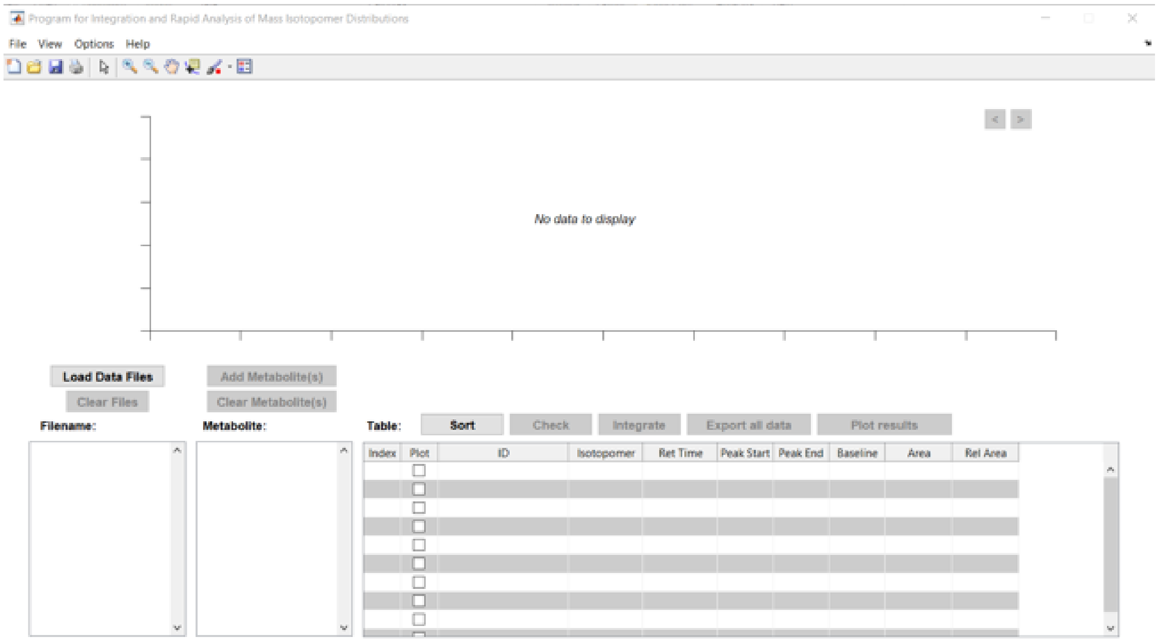

3. PIRAMID will estimate the resolution of the used equipment. However, it is advised that you input the resolution manually to ensure optimal results. To do that, click on “Set equipment resolution” on the View menu. The following GUI will appear, and you will be able to set the resolution as ppm or as a nominal mass.

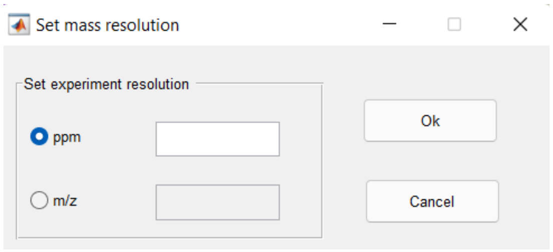

4. Click on “Load Data Files” and select a file to load. You must see the file in the files list and the corresponding chromatograms in the main table. The tool supports the following formats: .CDF, .netCDF, .mzml, and .mzxml.

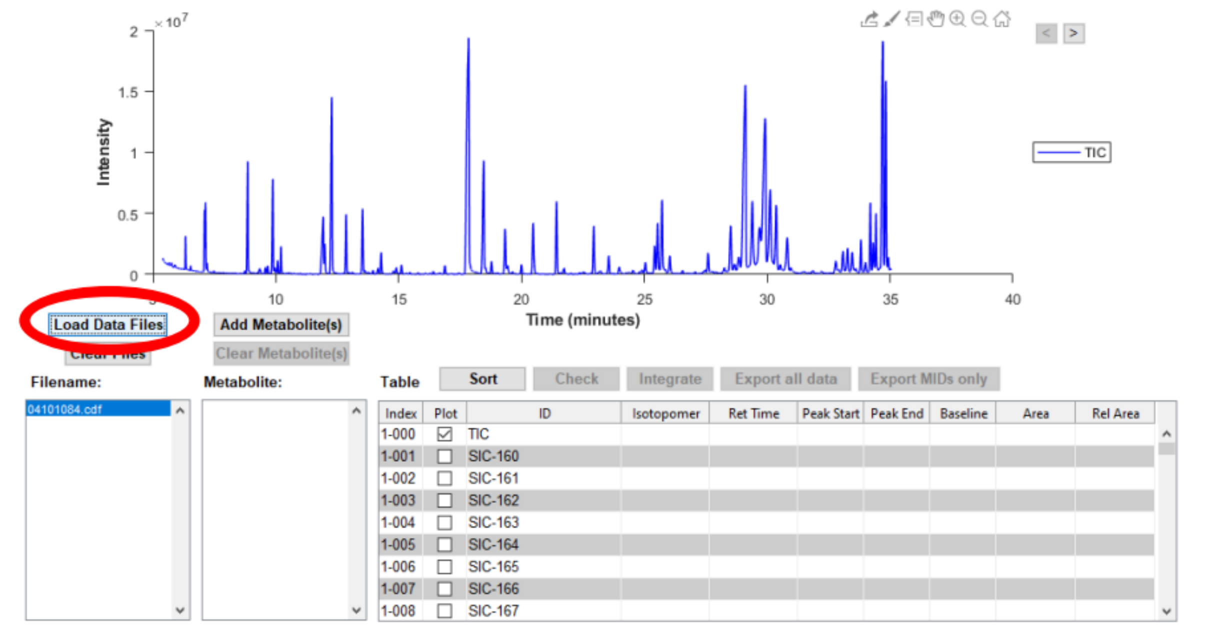

(Optional) If multiple files are loaded, a screen will ask you to input what the timepoints are and experimental groups (e.g. control vs experiment) for each one of the files. This step is needed to perform statistical analysis and to calculate the root mean square error (RMSE) of the samples. If this data is not input, these calculations will not be performed.

The panel where the timepoints and experimental groups can be changed can be accessed through the “Set timepoints panel” in the “View menu”

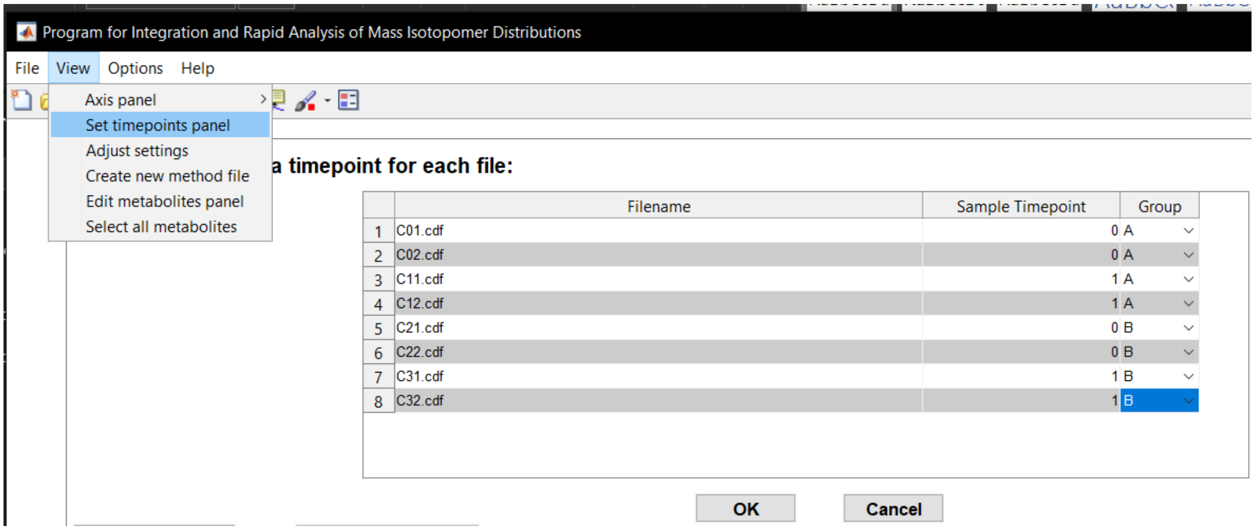

5. Click on “Add Metabolite(s)” and select the “Metabolite Mix.m” file that is attached. You must see the list of metabolites in the corresponding box.

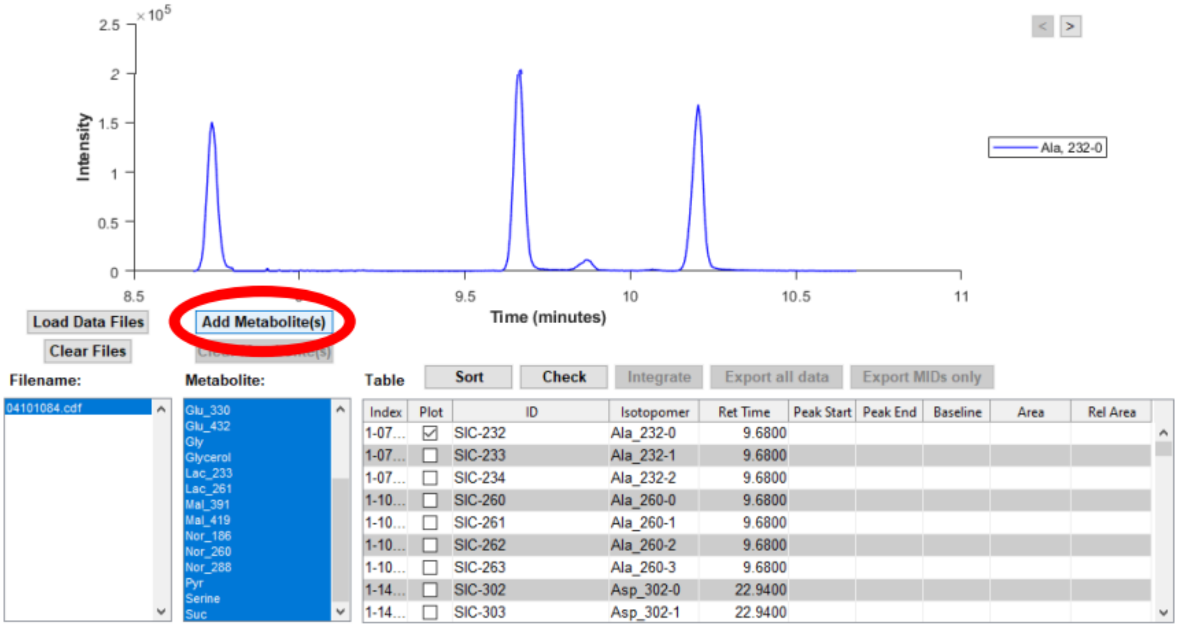

6. Select the metabolites from the list that you want to integrate and click on “Check”. You should see the changes of the retention time, peak start, peak end, and baseline reflected in the main table as well as vertical lines denoting the peak edges.

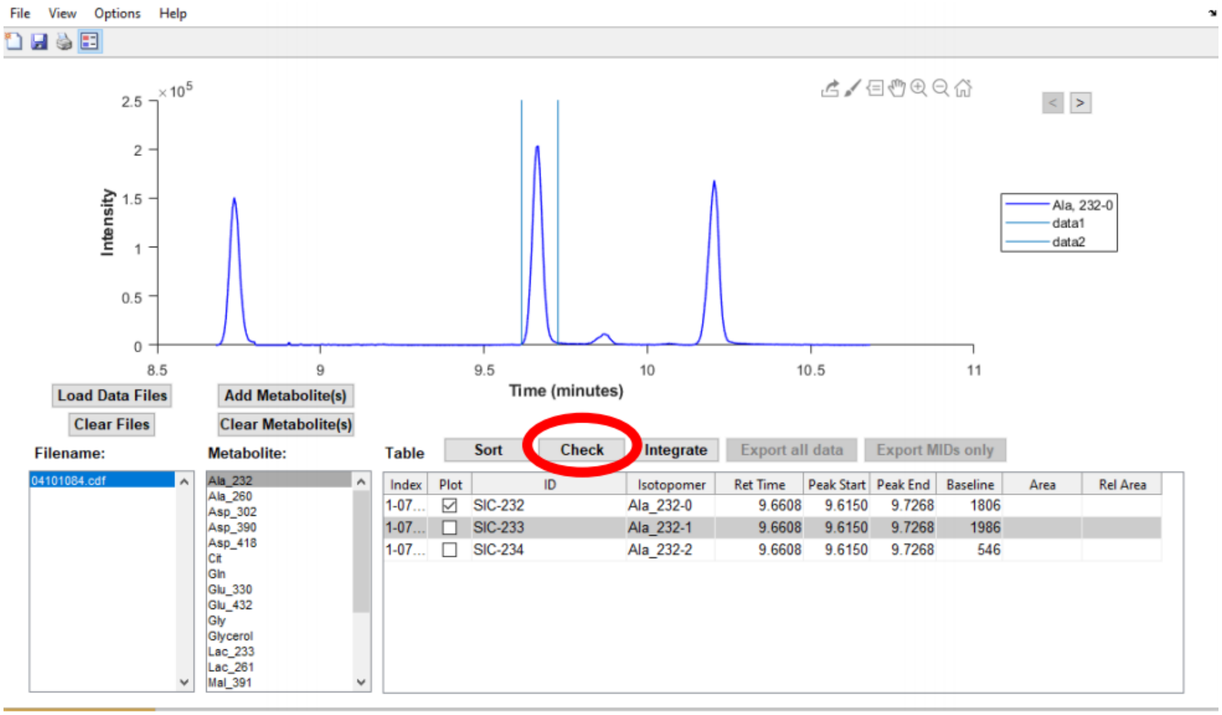

7. Click on “Integrate”. You should see the changes in the Area and Rel Area reflected in the main table.

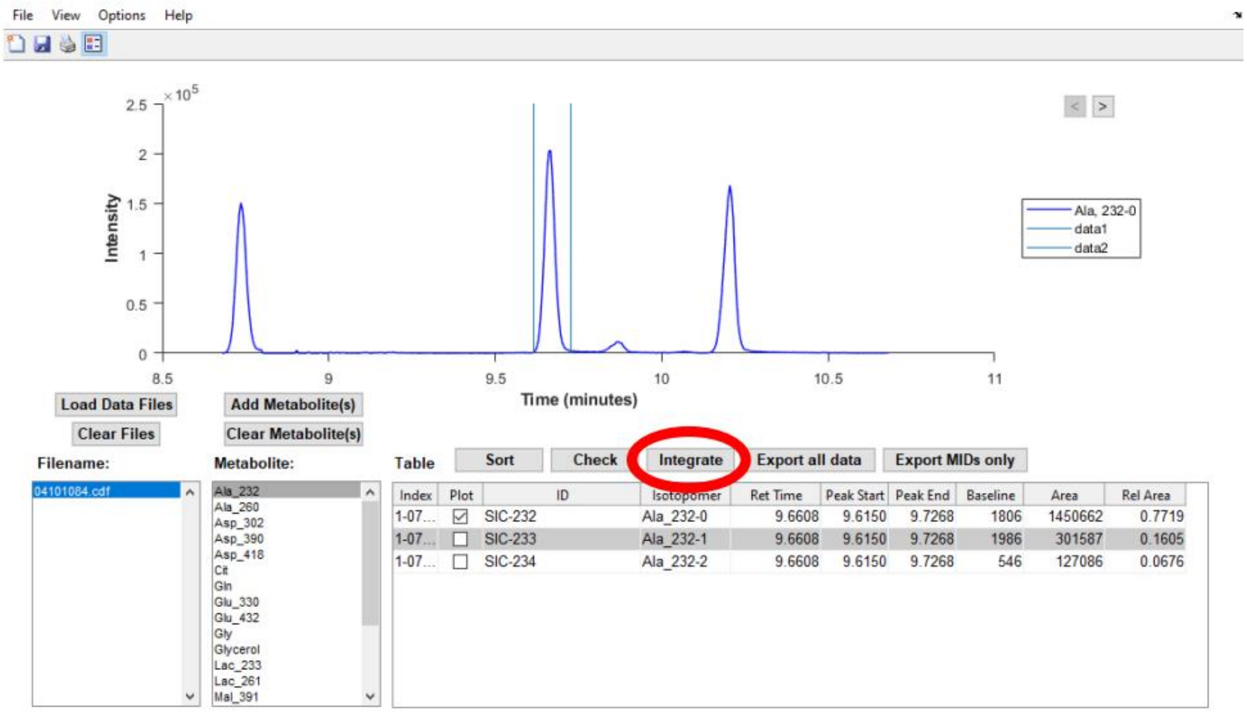

8. The relative abundance-corrected mass isotopomer distributions (MID) can be seen by clicking on “Correct for natural abundance”, under the Options menu.

9. Click on “Export all data” and select a folder where you want to save your file. A .csv file with the corresponding information should be exported.

B. Creating a new method file

1. Run the “Create_Method.m” file from the command window or from the editor. The following GUI should appear. Make sure that the files template.txt is in the same folder as the code.

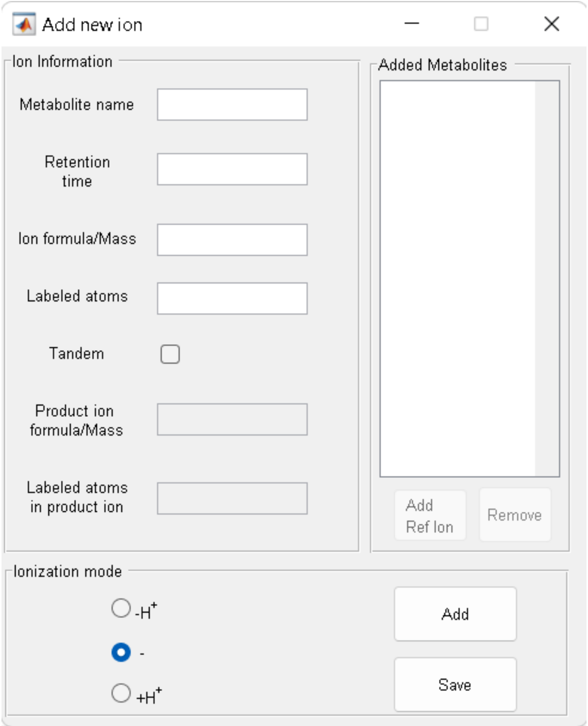

2. Add the Metabolite information.

1. Metabolite name: The name of the metabolite e.g. Pyruvate, Pyr, etc.
2. Retention time: The retention time of the metabolite in minutes e.g. 5.6
3. Ion formula/Mass: The chemical formula of the metabolite e.g. C6H12O3NSi. If the formula is unknown, the monoisotopic mass of the compound can be input.
4. Labeled Atoms: The number of mass isotopomers that will be analyzed and their corresponding labeled atom e.g. C3 to analyze 3 isotopomers from carbon.
   Only H, C, N, O, and S isotopes are supported.
5. Tandem: Only check if you want to analyze Tandem (MS2, MS/MS, MSMS) datasets.
6. If the equipment works by ionizing the fragments before analyzing them in the mass spectrometer, the corresponding ionization type can be selected. H^+ will add the mass of a hydrogen atom to the masses of the metabolites (Typically used when the mass spectrometers are run in positive mode), H^- will subtract the mass of a hydrogen atom (Typically used when the mass spectrometers are run in negative mode), and – will not alter the mass of the metabolites.
7. (Optional) If an additional reference ion is input to help the algorithm to select the best peak, click on "Add Ref Ion". The reference ions need to be input in a specific matrix form: The masses of the reference ions are input in a Nx2 matrix where N is the number of reference ions. The (N,1) position will mark the beginning of the reference range and the (N,2) position will mark the end of the range. The relative intensities of the reference ions need to be input as a Nx1 vector where the value in N will represent the relative intensity of the reference ion to the original metabolite. In the following example the sum of the intensities between the masses 174 and 179 are expected to show 70% of the intensity of the analyzed metabolite. Analogously the sum of intensities between the masses 217 and 220 are expected to show 130% of the intensity of the analyzed metabolite.

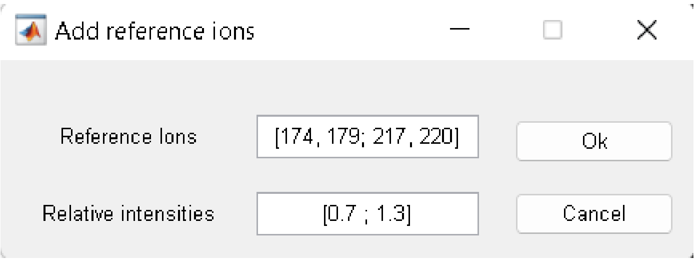

3. Click on Add. You should see your metabolite on the list in the right side.
4. If you need your file to have more metabolites, add them repeating step 2.
5. Once you are finished adding all the metabolites you need, click on Save.

* PIRAMID automatically calculates the mass isotopomers of a compound according to the information input in the “Ion formula” and “Labeled atoms” fields based on the possible combinations of the isotopes. However, if a metabolite containing only labeled atoms needs to be analyzed (e.g., as an internal standard) the isotopes can be input as follows:

**Isotope**	^{2}H	^{13}C	^{15}N	^{18}O	^{34}S
**Notation**	Hx	Cx	Nx	Ox	Sx

C. Modifying an existing method.

If an update of a method file is required, the following procedure should be followed.

1. Follow the steps 1-4 from User Guide A.
2. Select the “Edit metabolites panel” button in the “View” tab.

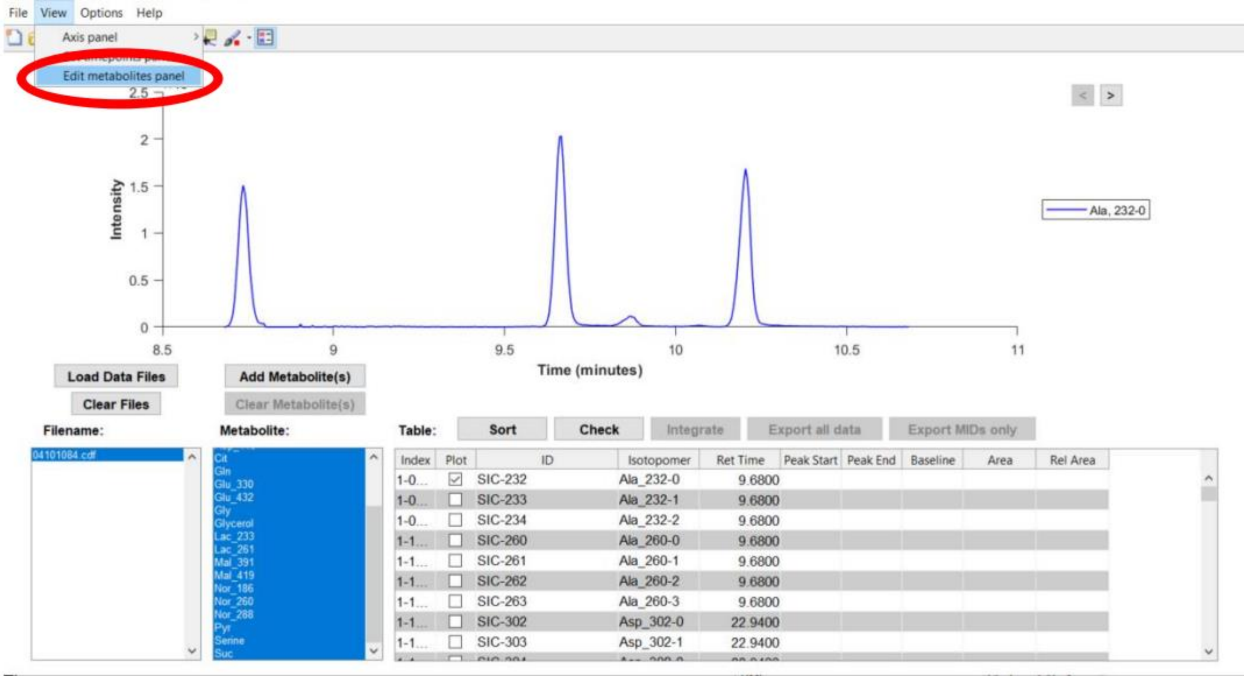

3. Select the metabolite you want to update

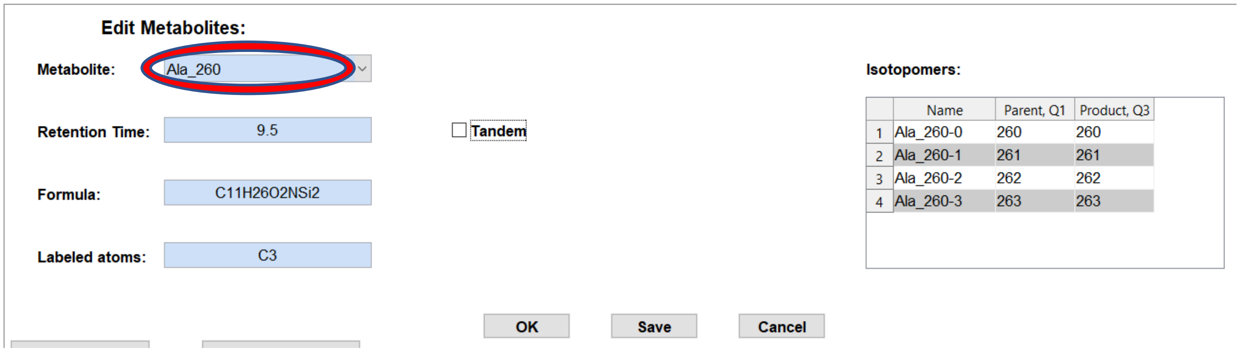

4. Input the changes in retention time, formula, names, or masses as needed.
5. Click on “OK” to save the changes for the current analysis or click on “Save” to modify the method file.

D. Performing a pairwise statistical analysis.

This feature is only available after setting experimental groups and timepoints (See section A.3) and integrating the peaks (See section A.6).

1. Select the “Perform statistical analysis” option under the Options tab.

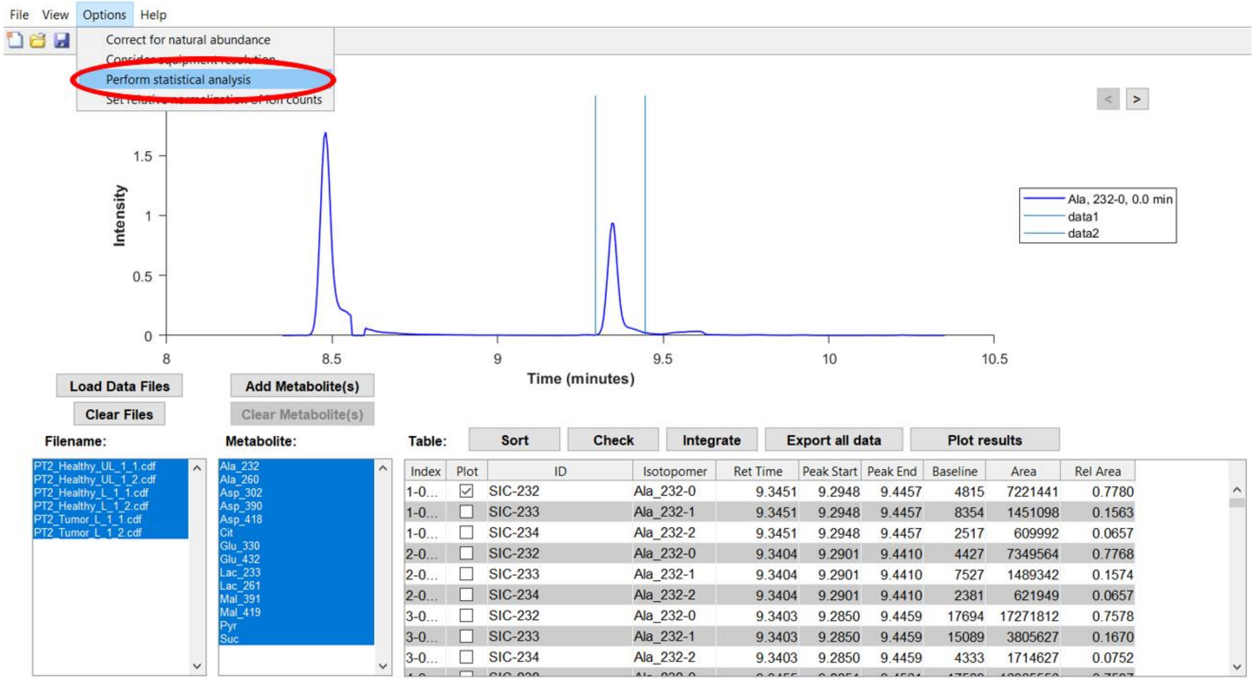

2. A GUI will appear asking to input the combinations of timepoints and experimental groups. Select the pairs that you want to compare.

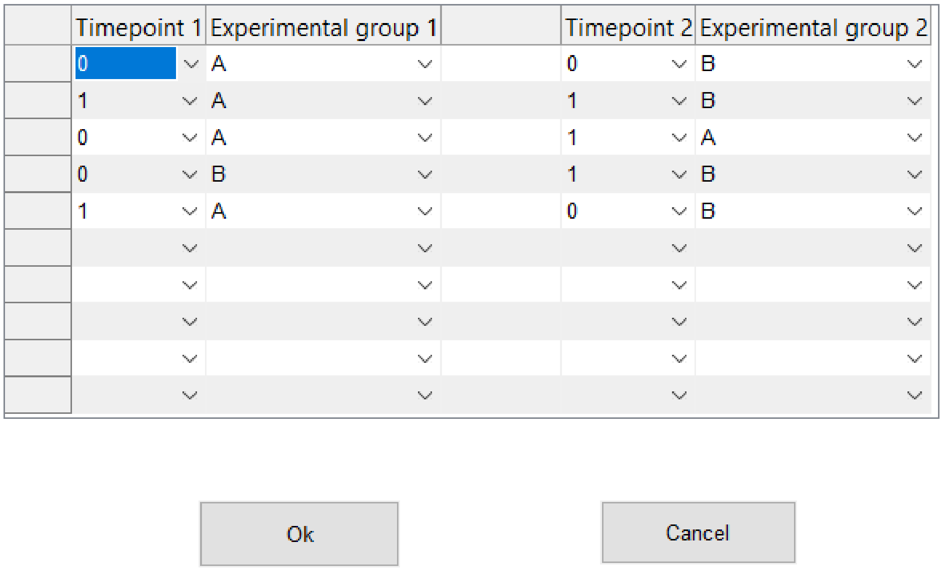

3. When exporting the results, a t-test will be performed between the input pairs and the hypothesis test result and the p-values will be exported in the selected file.

E. Plotting the results

This feature is only available after integrating the peaks (See section A.6). If timepoints and experimental groups are input (See section A.3) the plotted results will automatically be the means and standard deviations of the selected data.

1. After integrating the peaks, click on "Plot results"

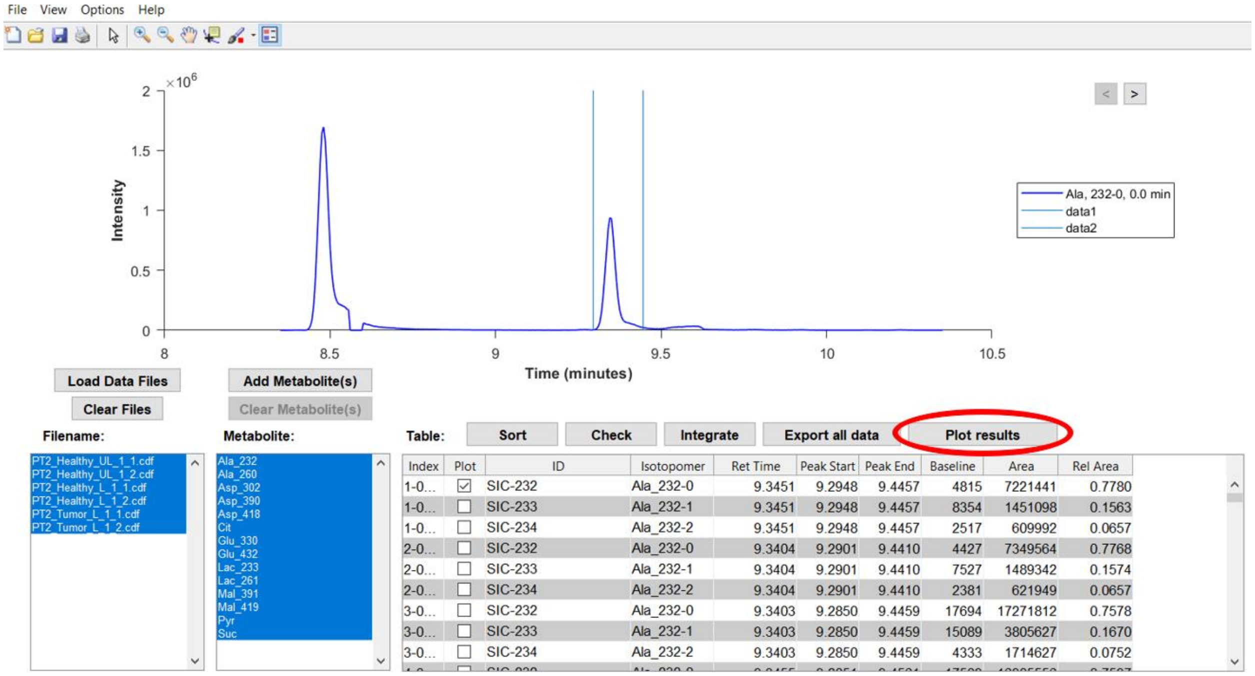

2. The following GUI will appear. The tool allows you to plot the MID of the integrated peaks in the form of a bar graph or their evolution in time in the form of a line graph.

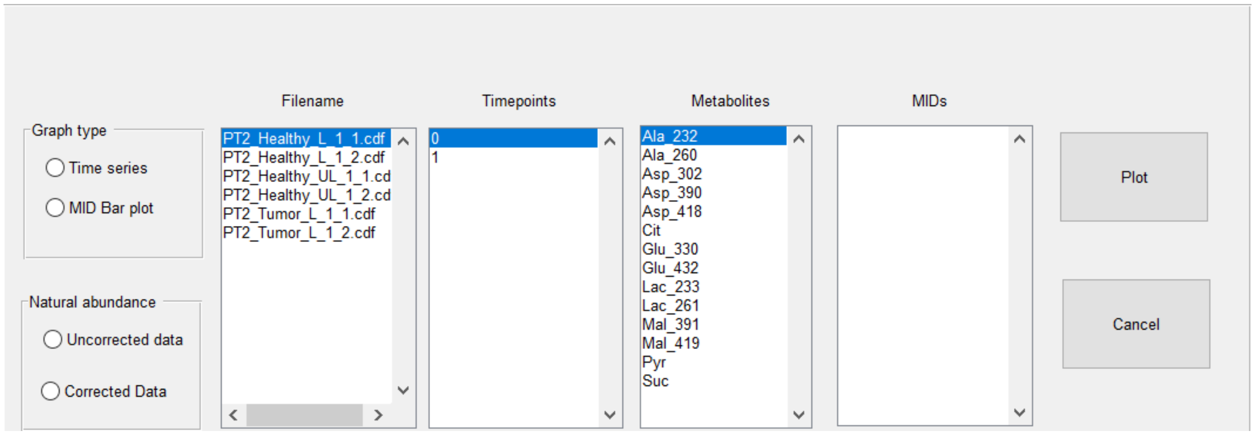

3. Select the type of graph, if the plotted data correspond to the natural abundance corrected data or not. Select the files, the timepoints, the metabolites, and the MIDs that you want to be plotted.
4. Click on Plot. Examples of each type of graph are shown below.

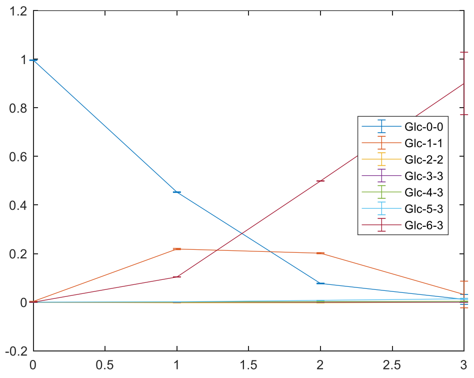

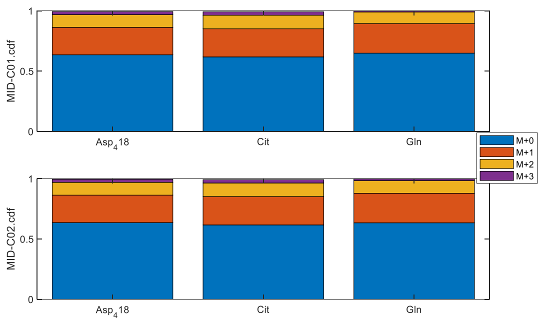

F. Normalize the enrichments to an internal standard.

Commonly some metabolomics protocols include the addition of an unlabeled standard with a known concentration to estimate the pool sizes of other metabolites. If this option is selected, an additional row in the results will be added normalizing the counts of all the metabolites to the selected one.

To do this, after integrating the data, click on “Set relative normalization of ion counts” in the Options menu and select the metabolite you want to use as a standard.

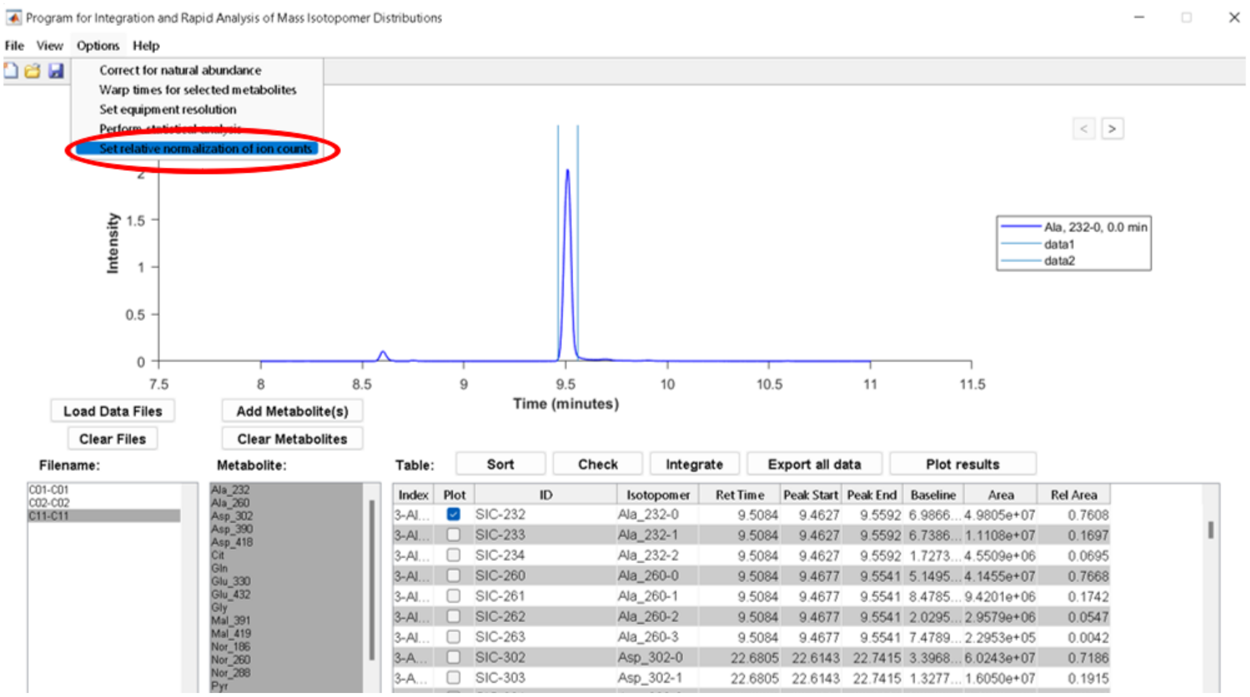

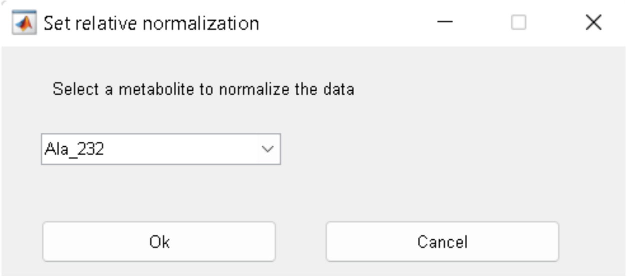

G. Correcting for retention times drifts.

To avoid adjusting manually the retention time of individual files that show drifts in the elution of the metabolites, a warping algorithm can be used.

In the following example, the monoisotopic masses of 4 different files (from different timepoints) show significant retention time drifts, which can be corrected by clicking on the button "Warp times for selected metabolites". This will align all the signals to the one in the first file of the list.

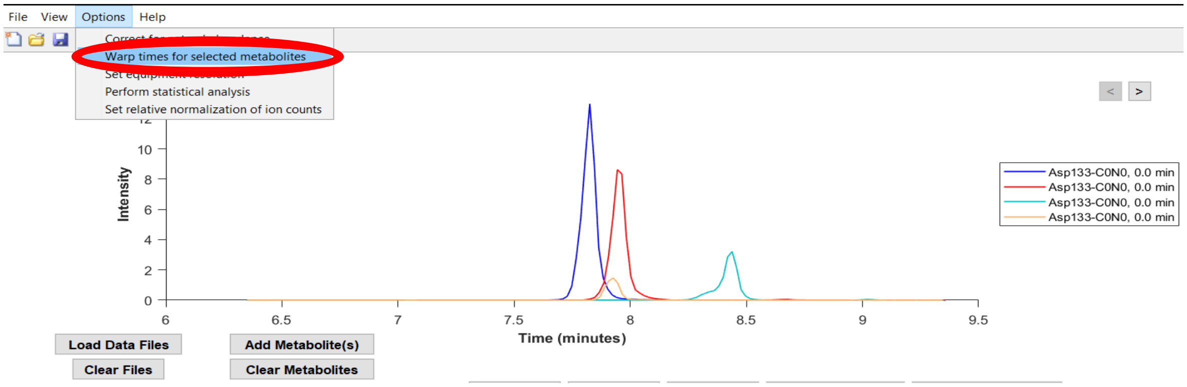

After clicking on the button, refresh the GUI by clicking on the metabolite. It can be seen that all the retention times were warped and can be integrated in batch using a single retention time.

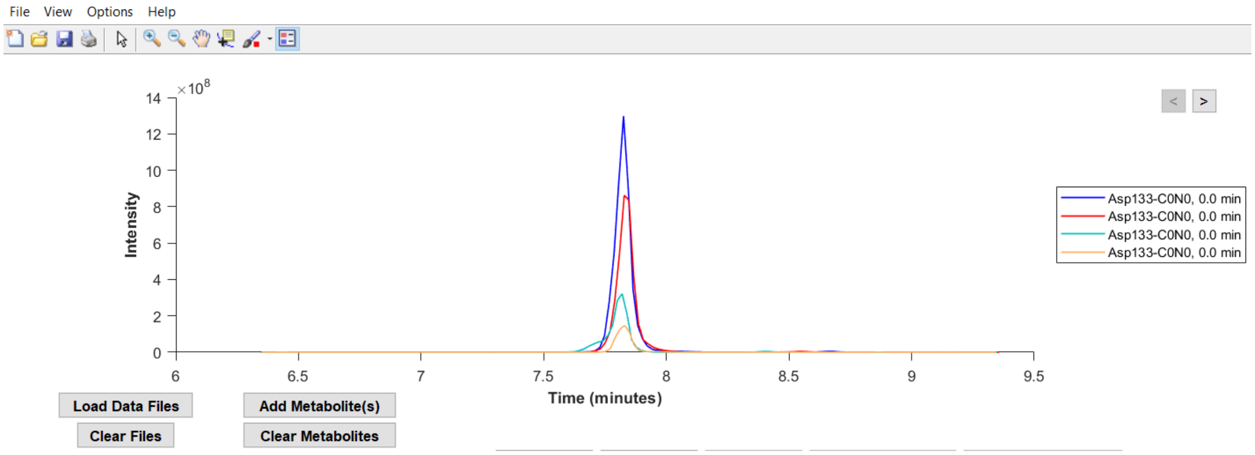

H. Adjusting advanced settings

If some of the default parameters used in the analysis need to be adjusted, it can be done by entering the settings panel by clicking on the "Adjust settings" button under the View menu.

The following parameters can be adjusted:

1. Window size [min]: After loading the metabolites, a frame of the chromatogram is extracted around the expected retention time. The extracted window size will be determined by this parameter. Default = 3.
2. Mass tolerance [ppm]: The mass tolerance that will be used by default when assigning the masses in the file to the target metabolites. Default = 10.
3. Baseline factor: Factor (N) used when determining the peak edges. The peak edges will be set once an intensity of N*(Baseline+Noise) is found. Default = 1.
4. Savitzky-Golay filter order: The order used when smoothing the signals using the SG filter. Default = 2.
5. Savitzky-Golay filter frame length: The frame length used when smoothing the signals using the SG filter. By default, this parameter is 0 which calculates the frame length based on the number of datapoints in the extracted chromatogram. Default = 0.
6. Second derivative baseline threshold: The normalized second derivative of the data needs to have a value below this value to be considered for a baseline. Default = 0.3
7. Carbon tracer purity: The purity of the carbon tracers used in the labeling. Default = 0.99
8. Non-carbon tracer purity: The purity of the non-carbon tracers used in the labeling. Default = 0.99
